# Pathogenicity and virulence of *Staphylococcus aureus*

**DOI:** 10.1080/21505594.2021.1878688

**Published:** 2021-01-31

**Authors:** Gordon Y. C. Cheung, Justin S. Bae, Michael Otto

**Affiliations:** Pathogen Molecular Genetics Section, Laboratory of Bacteriology, National Institute of Allergy and Infectious Diseases, U.S. National Institutes of Health, Bethesda, Maryland, USA

**Keywords:** *Staphylococcus aureus*, mrsa, immune evasion, neutrophils, biofilm, quorum-sensing, virulence, infection, toxins

## Abstract

*Staphylococcus aureus* is one of the most frequent worldwide causes of morbidity and mortality due to an infectious agent. This pathogen can cause a wide variety of diseases, ranging from moderately severe skin infections to fatal pneumonia and sepsis. Treatment of *S. aureus* infections is complicated by antibiotic resistance and a working vaccine is not available. There has been ongoing and increasing interest in the extraordinarily high number of toxins and other virulence determinants that *S. aureus* produces and how they impact disease. In this review, we will give an overview of how *S. aureus* initiates and maintains infection and discuss the main determinants involved. A more in-depth understanding of the function and contribution of *S. aureus* virulence determinants to *S. aureus* infection will enable us to develop anti-virulence strategies to counteract the lack of an anti-*S. aureus* vaccine and the ever-increasing shortage of working antibiotics against this important pathogen.

## Introduction

*Staphylococcus aureus* is one of the most infamous and widespread bacterial pathogens, causing a hard-to-estimate number of uncomplicated skin infections and probably hundreds of thousands to millions of more severe, invasive infections globally per year [1,2]. It is a leading causative agent in pneumonia and other respiratory tract infections, surgical site, prosthetic joint, and cardiovascular infections, as well as nosocomial bacteremia [3]. A review from 2012 estimated that *S. aureus* bacteremia has an incidence rate ranging from 20 to 50 cases/100,000 per year, and 10% to 30% of these patients will die from the infection [4]. In a more recent study from 2017, the annual number of deaths due to *S. aureus* bacteremia in the U.S. was reported to be 20,000 [5]. *S. aureus* bacteremia has been noted to account for a greater number of deaths than that caused by acquired immune deficiency syndrome (AIDS), tuberculosis, and viral hepatitis combined [1,4]. Other *S. aureus* infections, such as moderately severe skin infections, including furuncles, abscesses, and wound infections, are usually not life-threatening but may be accompanied by significant morbidity and pain. Due to their frequency (several millions annually in the U.S.), they represent a considerable public health burden [6]. Finally, *S. aureus* has also been associated with the development of atopic dermatitis [7].

*S. aureus* infections are particularly problematic due to frequently occurring antibiotic resistance in *S. aureus* isolates, among which methicillin-resistant *S. aureus* (MRSA) are the most important clinically [8]. Infections by MRSA are accompanied by increased mortality, morbidity, and hospital stay, as compared to those caused by methicillin-sensitive *S. aureus* (MSSA) [9]. The rates of methicillin resistance among clinical isolates varies greatly by country, ranging from single-digit rates in Scandinavian countries to over 50% for example in the U.S. and China [10]. While hospital-associated MRSA infections are on the decline in the U.S., Europe, China, and many other countries, likely due to increased hygiene and surveillance measures [5,11], they are still on the rise in poorly developed countries, for example in Africa [12]. In addition, even in the U.S., mortality caused by MRSA remains the highest for any antibiotic-resistant pathogen, reported by the CDC to be at ~20,000 in 2018 [5]. Furthermore, there is increased recognition of the considerable clinical importance of methicillin-sensitive *S. aureus* (MSSA) strains. Some MSSA lineages such as sequence type (ST) 398 can have high virulence, causing fatal infections [13,14]. MSSA infections are not monitored as closely as MRSA infections and recently implemented anti-MRSA measures did not cause a similar decrease in MSSA infections, as reported for example in the U.S. and U.K [15].

Resistance to other antibiotics is also widespread in *S. aureus*. For example, resistance to traditional beta-lactam antibiotics (penicillin and derivates) that are sensitive to beta-lactamase is virtually omnipresent in *S. aureus* [16]. Furthermore, *S. aureus* can show, often in combined form, resistance to almost all available antibiotics. Vancomycin remains the antibiotic of last resort for MRSA infections, with highly vancomycin-resistant strains (VRSA) having occurred but not spread, probably due to the strongly increased fitness cost that is imposed by vancomycin resistance genes [17]. However, there are strains that have acquired intermediate resistance to vancomycin (VISA) [18]. In addition to specific antibiotic resistance, nonspecific antibiotic resistance by biofilm formation plays a role in many *S. aureus* infections that are biofilm-associated [19]. These include prosthetic joint and all other indwelling medical device-associated infections, endocarditis, osteomyelitis, conjunctivitis, and others. Finally, mastitis is a prominent *S. aureus*-mediated, biofilm-associated infection in cattle that represents a major problem for milk and meat industries [20].

In contrast to many other bacterial pathogens, which often rely on only one or a few toxins to promote disease, *S. aureus* produces an astounding array of virulence factors. These include a plethora of toxins and immune evasion factors, and a vast array of protein and non-protein factors that enable host colonization during infection. While there has always been great interest in *S. aureus* virulence ever since this bacterium was first recognized as an important pathogen at the end of the 19^th^ century, recent developments have increased research efforts into unraveling *S. aureus* virulence mechanisms. These developments include first and foremost the rise in the early 2000s of community-associated (CA) MRSA, strains which combine methicillin resistance with high virulence potential in a previously unknown fashion [21], and the increasing recognition that highly virulent MSSA strains also represent a deadly threat [13,22]. The CA-MRSA pandemic in particular has initiated an immense research effort into toxins that attack white blood cells and which are widely believed to be associated with the epidemiological success of CA-MRSA [23].

In this review, we will give an overview of *S. aureus* virulence mechanisms. We will follow the definition describing virulence factors as those that promote establishment and maintenance of an infection by colonization and immune evasion mechanisms. Notably, we will not include mechanisms that contribute to asymptomatic colonization of *S. aureus* as a commensal, although – given that *S. aureus* infections commonly arise from this state – such mechanisms can be regarded as an important prerequisite for subsequent infection, and refer to dedicated reviews on the subject.

## Origins of infection

*S. aureus* infections usually originate from asymptomatic colonization or, probably more rarely and particularly in the hospital setting, from infected fomites or transfer from other individuals [24,25]. Several studies have reported associations of colonization of different body sites with invasive infection [24,26,27]. The nares are traditionally regarded as the main *S. aureus* colonization site, but *S. aureus* can colonize many skin sites in addition to the intestine. Persistent colonization only occurs in a subset of the population, ranging from ~10–30%, dependent on the particular study. Colonization of different body sites is usually highly correlated. This correlation is believed to originate from frequent touching and nose picking and the resulting distribution [28,29]. *S. aureus* may also be acquired from animals, especially in the livestock industry, where the development of livestock-associated MRSA (LA-MRSA) has been of great concern. However, outside of that setting, LA-MRSA strains are not considered major contributors to human MRSA infections [30].

**Figure 1.**
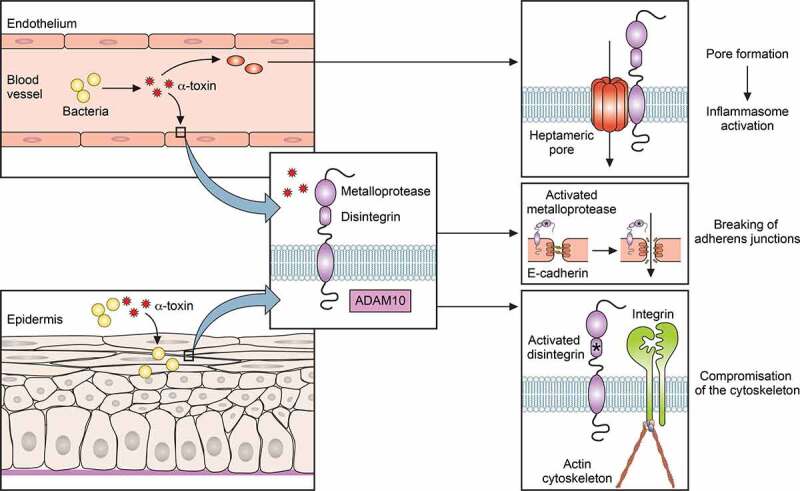
Role of α-toxin in *S. aureus* infection

Systemic *S. aureus* infection is always dependent on bacterial breach through the epithelial protective layer. For example, skin infections can develop from minor scratches of the skin and may become invasive [31]. However, *S. aureus* can also actively promote epithelial breach, for which α-toxin has predominantly been made responsible by its activation of the metalloproteinase domain-containing protein 10 (ADAM10) to cleave E-cadherin molecules [32,33]. This mechanism breaks adherens junctions and compromises the actin cytoskeleton [34] (Figure 1).

The major *S. aureus* toxin, α-toxin, works by two main mechanisms. Both are dependent on the ADAM10 receptor, which also contains metalloprotease and disintegrin domains. First, α-toxin causes pore formation in a series of target cells via formation of a heptameric pore. Second, it causes epithelial and endothelial breach via breaking adherens junctions and compromising the cytoskeleton.

The contamination of indwelling medical devices represents another route of infection that occurs frequently in the hospital setting. The main mechanism underlying this infection route is the capacity of *S.aureus* to adhere to the devices’ plastic material as well as to the matrix molecules that cover the devices soon after insertion, and to form a biofilm on the device [19]. Biofilm formation is also the suspected cause for menstrual staphylococcal toxic shock syndrome (TSS), in which specific *S. aureus* strains producing toxic shock syndrome toxin-1 (TSST-1) form biofilms on high-absorbency tampons [35].

Food poisoning is a special case of acute *S. aureus* infection in which contaminated foods containing staphylococcal enterotoxins (SEs) are ingested [36]. SEs cause emesis in a not completely understood manner that involves induction of histamine release from intestinal mast cells [37]. Similar to TSST-1, they are superantigenic toxins, which activate T-cells in a predominantly nonspecific manner, resulting in an excessive immune response that includes polyclonal T cell activation and massive cytokine release. Systemic infections originating from acute staphylococcal food poisoning are very rare. Whether sustained intestinal colonization by *S. aureus* can lead to gastro-intestinal and even systemic disease is not known. Probably, the observed correlation between intestinal *S. aureus* colonization and other forms of *S. aureus* infection rather stems from the intestine representing a reservoir for the distribution of *S. aureus* to other epithelial colonization sites [26].

Finally, *S. aureus* also can make use in an opportunistic fashion of primary harm done by other pathogens or predisposing conditions. This occurs, for example, in lung infections that have been initiated by a viral infection such as the flu, in which *S. aureus* secondary infection is often the ultimate cause for death [38,39]. Furthermore, *S. aureus* has been shown to contribute to the development of atopic dermatitis via specific toxins, including δ-toxin or similar cytolytic peptides called phenol-soluble modulins (PSMs) by activating mast cells [7,40,41]. Moreover, *S. aureus* can complicate skin infections caused by other pathogens. One such example is the exacerbation of Buruli ulcers even after the original pathogen (*Mycobacterium ulcerans* in this case) has been eradicated by antibiotic treatment [42].

## Establishment of infection

**Figure 2.**
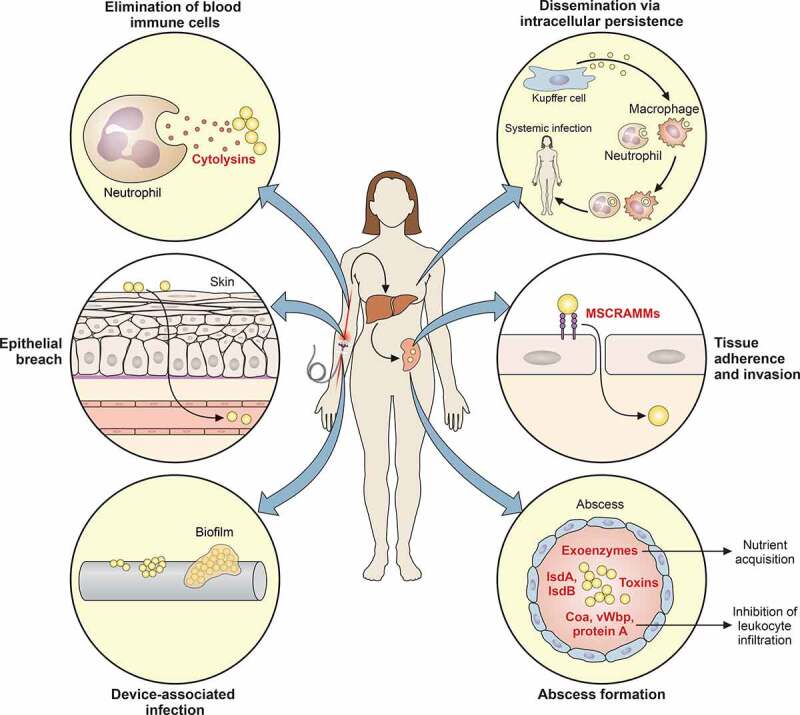
Stages of *S. aureus* systemic infection

After epithelial breach and systemic invasion (Figure 2), the success of a staphylococcal infection depends on the effective evasion of host defenses. *S. aureus* accomplishes this by leaving the bloodstream with its high concentration of cellular and humoral immune defense mechanisms to invade organs and tissues, where it forms encapsulated abscesses. In the case of skin or lung infections, abscesses can form directly after epithelial breach.

*S. aureus* systemic infection frequently begins with a breach of the bacteria through the protective barrier of the skin or dissemination from a biofilm that can form on indwelling medical devices. In the bloodstream, the bacteria can actively attack and eliminate immune cells such as neutrophils via cytolytic toxins, or – alternatively – persist in such cells to achieve systemic distribution. Passage through the liver, where the bacteria are confronted by the phagocytic activity of Kupffer cells, represents a bottleneck for subsequent systemic infection. If the bacteria survive this stage, they can further distribute through the bloodstream and attach to and invade tissue cells, which is mediated by MSCRAMM surface proteins. Subsequent abscess formation is impacted by many different bacterial factors that include specific surface proteins, toxins, and exoenzymes.

While still in the blood, *S. aureus* uses an astounding series of mechanisms to avoid elimination. These range from toxins that destroy phagocytes (leukocidins) and mechanisms that trigger phagocyte apoptosis to the inhibition of complement factors, in addition to agglutination and the formation of thrombi. We will discuss in detail the *S. aureus* factors underlying these immune evasion mechanisms below.

## Avoidance of killing by phagocytes

Neutrophils are the most prominent leukocytes in the blood, representing ~60% of the leukocyte population. They play a major role in controlling *S. aureus* infection, as evidenced by the extreme susceptibility to *S. aureus* infection observed in patients with neutrophil defects [43]. In addition, recent research has attributed an important role to liver Kupffer cells in *S. aureus* infection, as pathogen elimination in the liver by those cells has been described as a key initial bottleneck for the development of subsequent *S. aureus* bacteremia and the establishment of infection in other organs [44,45]. Most mechanisms of *S. aureus* evasion of phagocyte killing have, however, been investigated in neutrophils.

**Figure 3.**
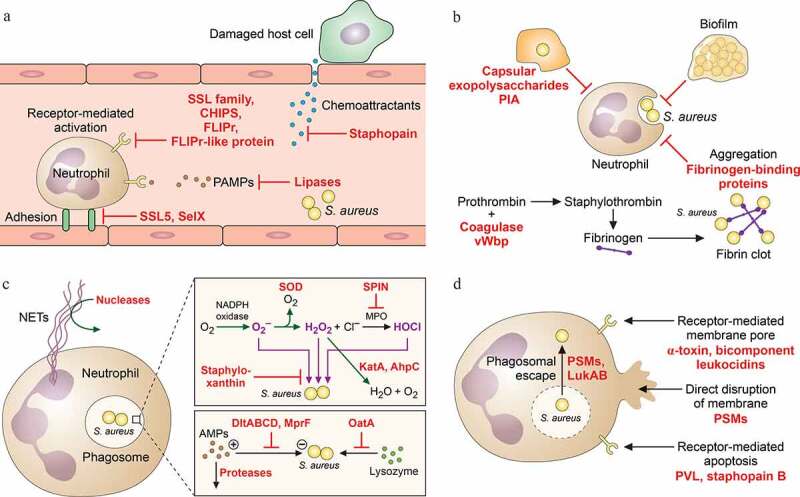
*S. aureus* mechanisms of immune evasion

*S. aureus* avoids being eliminated by neutrophils on many levels that include 1) the inhibition of neutrophil extravasation from the bloodstream into the tissues, neutrophil activation, and chemotaxis, 2) inhibition of phagocytosis by aggregation, protective surface structures, and biofilm formation, 3) inhibition of opsonization, 4) inhibition of neutrophil killing mechanisms, and 5) direct elimination of neutrophils by cytolytic toxins or triggering of apoptosis [46,47]. It has been argued that particularly the efficacy of the latter two mechanisms, which are independent of whether opsonization by antibodies and other opsonins has been successful, may underlie the difficulties of finding a working vaccine for *S. aureus*, which still remains elusive [48,49] (Figure 3).

*S. aureus* inhibits elimination by mechanisms of – mostly, innate – host defense on many different levels, including inhibition of immune recognition/opsonization (A), aggregation/matrix production (B), resistance to phagosomal bactericidal mechanisms (C), and direct elimination of phagocytes and other immune cells (D). (A) *S. aureus* secretes a plethora of molecules that inhibit opsonization and complement activation. It also inhibits adhesion of neutrophils to the vascular endothelium, inhibiting neutrophil extravasation from blood vessels into infected tissues. Finally, *S. aureus* lipase decreases the pro-inflammatory activity of lipoprotein pathogen-associated molecular patterns (PAMPs). (B) *S. aureus* produces extracellular substances, such as capsule or the exopolysaccharide PIA, which inhibit phagocytosis. Furthermore, many strains produce biofilms, which also provides for resistance to phagocyte attacks. Finally, *S. aureus* produces factors that lead to fibrin clots and fibrin/bacterial aggregates. (C) *S. aureus* efficiently subverts both oxygen-dependent and -independent neutrophil bactericidal mechanisms. Superoxide dismutase (SOD) converts harmful superoxide and catalase as well as AhpC convert hydrogen peroxide to innocuous molecules. The SPIN protein inhibits myeloperoxidase (MPO), which produces the most potent reactive oxygen species (ROS), hypochlorite. The yellow pigment staphyloxanthin generally protects *S. aureus* cells from ROS. The activity of antimicrobial peptides (AMPs) is diminished by secreted proteases and reducing the anionic character of the bacterial cell surface. Lysozyme activity is reduced by OatA-catalyzed alteration of peptidoglycan. Finally, *S. aureus* secretes nuclease that can digest neutrophil extracellular traps (NETs). (D) *S. aureus* produces several cytolysins including bicomponent leukocidins, α-toxin, and phenol-soluble modulins (PSMs) that directly lyse leukocytes, some of which have also been shown to cause phagosomal escape/lysis after phagocytosis. PSMs and α-toxin also lyse other cell types. Furthermore, some secreted *S. aureus* molecules can trigger receptor-mediated apoptosis.

### Inhibition of neutrophil extravasation, activation, and chemotaxis

Unless neutrophils directly interact with pathogens in the bloodstream, they need to migrate from the blood to the site of infection, which is a complex process that involves neutrophil rolling and adhesion to the endothelium, penetration through the endothelium (diapedesis), as well as activation and migration to the pathogen along a gradient of chemoattractants in a process called chemotaxis [50]. Activation occurs via primary stimulation (“priming”) by specific cytokines such as IL-8, CSF, and IFN- γ or the C3a and C5a complement factors [51]. Chemoattractants can be host-derived, such as leukotriene B4 or IL-8, or produced by the bacteria. In Gram-positive bacteria, neutrophil activators/chemoattractants include the specific N-terminal lipoylated structure of lipoproteins [52], peptidoglycan [53], unmethylated CpG sequences in DNA [54], and formylated peptides, which derive from the N-formylation of methionine during protein synthesis [55]. Lipoteichoic acid is also pro-inflammatory, but its role may have been considerably overestimated as a result of contamination with lipopeptides [56]. Importantly, all these structures are specific for bacteria and thus enable the immune system to recognize bacterial invaders. They have been termed “pathogen-associated molecular patterns” (PAMPs) and usually activate Toll-like receptors (TLRs) [57], which function as homo- or heterodimers. Diacylated lipoproteins activate TLR2/TLR6, while triacylated lipoproteins activate TLR2/TLR1 and CpG sequences TLR9. In *S. aureus*, the PSMs, which include the δ-toxin, form a large part of the secreted protein amount and, being secreted without a signal peptide, retain their pro-inflammatory N-formyl methionine part [58,59]. However, PSMs are also chemotactic for neutrophils without N-formylation and both the N-formylated and non-N-formylated forms predominantly activate the formyl peptide receptor 2 (FPR2) [60]. Classical PAMPs are not secreted and require direct surface interaction or shedding from the surface to act as activators or chemoattractants. Interestingly, PSMs facilitate shedding of lipoproteins, which are possibly the most important *S. aureus* chemoattractants [56], thus having a double direct and indirect impact on neutrophil attraction and activation [61].

*S. aureus* inhibits neutrophil extravasation, activation, and chemotaxis using a plethora of factors and mechanisms. The staphylococcal superantigen-like protein 5 (SSL5) and SelX, which bind to P-selectin glycoprotein ligand-1 (PSGL-1) on the leukocyte surface, inhibit extravasation via the inhibition of neutrophil adhesion via PSGL-1 to the P-selectin anchor on endothelial cells [62]. SelX has been found to significantly contribute to pathogenesis in a rabbit pneumonia model via neutrophil inhibition rather than its superantigenic activity [63]. Activation and chemotaxis are inhibited by many members of the SSL family including SSL3, SSL4, SSL5, and SSL10, which bind to G protein-coupled receptors (GPCRs) and TLRs. For example, SSL3 and SSL4 bind to TLR2 [64,65], SSL10 to CXCR4 [66], and SSL5 generally to GPCRs [67]. CHIPS (chemotaxis-inhibitory protein of *Staphylococcus*) binds and inhibits FPR1 and C5aR [68,69], while FLIPr and FLIPr-like inhibit FPR1 and FPR2 [70,71]. The secreted *S. aureus* staphopain protease and lipase also contribute to the inhibition of chemotaxis. Staphopain degrades CXCR2, thereby inhibiting neutrophil migration toward cytokines recognized by that receptor [72]. The Geh lipase has recently been discovered to degrade lipoproteins by removing the pro-inflammatory lipoylated N-terminus, preventing recognition of these PAMPs by neutrophils [73].

While the importance of pathogen recognition for example via TLR2 is reflected by the increased disease manifestations found in TLR2 knockout mice infected with *S. aureus* [74], the contribution to pathogenesis of the many neutrophil chemotaxis and activation-inhibitory factors is hard to monitor in mouse models due to their functional redundancy and specificity for humans. In many cases, their relative contribution to *S. aureus* virulence is therefore not known.

### Inhibition of phagocytosis by aggregation, protective surface structures, and biofilm formation

Many invasive microorganisms produce capsular exopolysaccharides whose main function is to protect the pathogen from phagocytosis. Several clinical strains of *S. aureus* produce capsules, commonly of serotypes 5 or 8 with the structure (→4)-3-O-Ac-β-d-ManNAcA-(1→4)-α-l-FucNAc-(1→3)-β-d-FucNAc-(1→)*_n_* and (→3)-4-O-Ac-β-d-ManNAcA-(1→3)-α-l-FucNAc-(1→3)-β-d-FucNAc-(1→)*_n_*, respectively [75,76]. However, the USA300 lineage, which has become the main source of hospital- and community-associated infections in the U.S [1,77]., does not produce a capsule, indicating that other immune evasion mechanisms can substitute for the protective function of capsule [78]. This situation is particularly important for the design of anti-*S. aureus* vaccines, which in the past have frequently relied on capsular polysaccharides 5 and 8 as components [79]. Interestingly, capsule formation is only associated with increased virulence in some infection types, such as bacteremia, while it appears counterproductive in situations where it may shield necessary adhesins, such as endocarditis [80,81].

Polysaccharide intercellular adhesin (PIA, also called PNAG for poly-N-acetyl glucosamine according to its structure) is a cell surface-located homopolymeric exopolysaccharide of *S. aureus* and other staphylococci that is made of partially deacetylated GlcNAc units [82,83]. The positive charge that is introduced by deacetylation attracts the molecule to the negatively charged bacterial surface [84,85]. PIA/PNAG is a major component of the staphylococcal biofilm matrix [19]. With biofilm formation by itself representing an efficacious mechanism to inhibit phagocytosis, PIA/PNAG prevents phagocytosis by two mechanisms: (i) shielding the cellular surface from phagocyte attacks and (ii) contributing to the biofilm network [85,86]. Similar to capsule, PIA/PNAG is not produced in all *S. aureus* strains, emphasizing the multi-factorial character of phagocyte evasion mechanisms [87]. Of note, PIA/PNAG has also been investigated as a vaccine component [88].

While biofilm formation most frequently occurs on indwelling medical devices and refers to surface-associated bacterial agglomerations, *S. aureus* uses other aggregation mechanisms in the blood to escape ingestion by phagocytes. Namely, it produces thrombi by the combined, non-redundant activity of coagulase and von Willebrand factor-binding protein (vWbp), which bind prothrombin (factor II of the coagulation cascade), forming a complex called staphylothrombin [89,90]. Staphylothrombin can cleave fibrinogen to from fibrin clots in the absence of the vascular damage signal that is normally necessary for this step. *S. aureus* then uses fibrinogen-binding proteins such as clumping factor A (ClfA) to adhere to the fibrin clots and form fibrin-containing bacterial aggregates [91]. The fibronectin-binding proteins FnBPA and FnBPB also activate aggregation of platelets [92].

### Inhibition of opsonization

Efficient phagocytosis requires opsonization of the bacterial targets by antibodies (immunoglobulins, Igs) or complement. Igs bind to pathogens via their F_ab_ segments and to phagocytes via their F_c_ regions. There are differences in opsonization efficacy depending on the Ig subclass, with IgM having the highest due to its polymeric structure. In addition to being opsonic, the presence of Igs on the bacterial cell surface stimulates the classical pathway of complement fixation.

*S. aureus* produces three proteins that interfere with Ig deposition. The best-known is surface protein A (SpA), which produces a “camouflage coat” of nonspecific Igs on the *S. aureus* surface via nonspecific binding to the F_c_ regions of IgG [93]. It also binds to the F_ab_ region of IgM, serving as a B cell superantigen and causing B cell apoptosis. Furthermore, it skews the immune response away from other *S. aureus* virulence factors by triggering the production of plasma B cells that recognize almost exclusively protein A [94,95]. Sbi (*S. aureus* binder of IgG) binds exclusively to the F_c_ region of IgG, but also to the serum component apolipoprotein H, in addition to complement factors H and C3 [96,97]. SSL10, a protein with multiple further functions in immune evasion, also binds to the IgG F_c_ region, preventing receptor-mediated phagocytosis [98].

The primary role of the complement system during infection with Gram-positive bacteria such as *S. aureus* is the deposition of C3b on the bacterial surface for opsonization, which happens when C3 is cleaved via C3 convertases from one of three independent pathways (the lectin classical, and alternative pathways) [99]. Further complement factors, such as C5a that is formed upon C3-C3b interaction, act as chemoattractants for additional immune cells, a mechanism reported to also matter during *S. aureus* systemic infection [100].

*S. aureus* produces a plethora of proteins that inhibit the complement system. The most central and versatile one, staphylococcal complement inhibitor (SCIN), inhibits all three pathways by a multi-pronged approach that leads to the inhibition of C3 convertases, diminishing C3b deposition and C5a chemoattractant formation [101]. Many further *S. aureus* virulence factors with other previously identified roles in pathogenesis also inhibit complement. For example, the extracellular fibrinogen-binding protein Efb binds C3 via its C-terminus and fibrinogen via its N-terminus, thereby covering bacteria in a surface layer of fibrinogen that inhibits recognition of surface-bound C3b [102]. The homologous extracellular complement-binding protein Ecb lacks fibrinogen-binding activity but inhibits the alternative pathway C3 convertase and all C5 convertases [103]. Several further *S. aureus* proteins inhibit specific pathways: the collagen adhesin (Cna) the classical pathway [104], SdrE the alternative pathway [105,106], and the extracellular adherence protein (Eap) the lectin and classical pathway [107]. Finally, SSL7 inhibits complement in two ways, via inhibition of IgA recognition by binding to the F_c_ region of IgA and by binding to C5 [108].

In addition to these very specific opsonization inhibition mechanisms, opsonization can be inhibited via the proteolytic activity of secreted *S. aureus* proteases. *S. aureus* produces a series of secreted proteases with relatively low substrate specificity, the most important of which are staphylococcal serine protease (V8 protease; SspA), cysteine protease (SspB), metalloprotease (aureolysin; Aur), and staphopain (Scp) [109]. While the main function of these proteases may consist in nutrient acquisition, it is likely that they also destroy many immune defense proteins. This has specifically been shown in the case of complement for aureolysin, which cleaves C3 [110]. Furthermore, the V8 protease has been shown to cleave Igs [111]. However, whether this mechanism contributes to pathogenesis is largely speculative [112].

Of note, many complement inhibitory factors of *S. aureus* are human-specific; therefore, their contribution to virulence is hard to model in mouse infection models [46]. Furthermore, for many of the abovementioned factors – such as the proteases – the specific contribution to complement-related pathogenesis is hard to determine due to their multi-functional nature. However, where such a measurement is possible and has been performed, for example measuring in-vivo mortality and neutrophil influx using an *efb*/*ecb* double mutant, it showed a key role of complement inhibition in *S. aureus* virulence that adds to the evolution biology argument that many such mechanisms are conserved in clinical *S. aureus* isolates [113].

### Inhibition of neutrophil killing mechanisms

Once neutrophils have managed to ingest *S. aureus* despite the many mechanisms *S. aureus* has to evade phagocytosis, *S. aureus* cells are attacked by the very efficient bactericidal activities that are present in the neutrophil phagosome. Myeloperoxidase (MPO), which is released from primary granules, produces reactive oxygen species (ROS). Primary granules also release antimicrobial peptides (AMPs) such as defensins. Secondary granules release further antimicrobial proteins, such as lysozyme. The bactericidal mechanisms of a neutrophil are commonly categorized as oxygen-dependent (myeloperoxidase, MPO) and -independent (antimicrobial peptides and proteins) [114]. *S. aureus* has developed many mechanisms to interfere with both.

The oxygen-dependent mechanisms consist of NADPH oxidase, which produces superoxide from O_2_. Superoxide (O_2_^−^) is then spontaneously converted to hydrogen peroxide (H_2_O_2_). MPO produces hypochlorous acid (HOCl) from the reaction of H_2_O_2_ with chloride. HOCl is the major ROS effector molecule [115]. *S. aureus* has several mechanisms providing resistance to ROS and inhibiting ROS production enzymes. Staphyloxanthin is the orange pigment that has given *S. aureus* its name (“aureus,” golden). This molecule contains a series of conjugated double bonds that scavenge radicals originating from ROS activity [116,117]. Additionally, *S. aureus* produces superoxide dismutase, which converts superoxide to the less toxic H_2_O_2_ [118,119], as well as catalase (KatA) and alkyl hydroperoxide reductase C (AhpC), which further detoxify H_2_O_2_ by turning it into oxygen and water [119,120]. Furthermore, *S. aureus* produces a lactate dehydrogenase, which is inducible by nitric oxide (NO), another ROS, and contributes to maintaining redox homeostasis in the phagosome [121]. Also, *S. aureus* can inhibit the oxidate burst by converting ADP and AMP to adenosine [122] and resist the toxicity of copper, which is imported into macrophage phagosomes and contributes to ROS production, via a mobile genetic element-encoded copper hypertolerance system [123]. Lastly, staphylococcal peroxidase inhibitor (SPIN) directly inhibits MPO [124].

Defensins and other AMPs are commonly positively charged and many function as pore formers in the bacterial membrane. With AMPs being attracted to the bacterial surface by electrostatic interaction, staphylococcal resistance mechanisms consist in reducing the negative charge of the membrane and cell wall [125]. The *dlt* operon esterifies hydroxyl groups in teichoic acids with alanyl residues, introducing one positive charge per alanine into this cell surface polymer, increasing the net charge of the cell surface [126]. The MprF (Multiple peptide resistance factor) membrane enzyme is a lysyl-phosphatidylglycerol (Lys-PG) synthase and Lys-PG flippase that introduces Lys-PG in the outer layer of the cytoplasmic membrane, also reducing attraction of AMPs [127,128]. Both the *mprF* and *dlt* genes are regulated by a system termed antimicrobial peptide-sensing system (Aps)RSX, also called (Gra)RSX (for gramicidin resistance), that responds to binding of cationic AMPs [129]. ApsRS/GraRS form a two-component system. ApsX/GraX and the ApsRSX/GraRSX-controlled VraFEG transport system are also involved in signal transduction by that system in a way that is not completely understood [130–132]. AMPs are also subject to proteolytic degradation by secreted *S. aureus* proteases, a mechanism that is also induced by and affects the negatively charged AMP, dermcidin [133]. Among the many antibacterial proteins neutrophils secrete into the phagosome, lysozyme is knowingly very efficient against Gram-positive bacteria. However, its efficacy toward *S. aureus* is limited due to the enzymatic activity of an enzyme called OatA, which acetylates the muramic acid parts of peptidoglycan [134]. On the other hand, *S. aureus* subverts the activity of antimicrobial proteases secreted into the neutrophil phagosome via the extracellular adherence protein (Eap) [135].

Neutrophil extracellular traps (NETs), another, unconventional mechanism of pathogen killing that neutrophils use after pathogen-induced lysis (“NETosis”) [136], is also believed to contribute to host defense against *S. aureus*, although this is controversial [137]. The finding that secreted *S. aureus* nuclease protects from NET-mediated killing in vitro and contributes to infection in vivo indicates that there may be a role for NETs in fighting *S. aureus* infections [138], although this is difficult to demonstrate directly. In addition, *S. aureus* produces an enzyme called AdsA that produces deoxyadenosine from nuclease-digested NET DNA [139]. Deoxyadenosine triggers caspase-3–mediated death of other immune cells, which has been shown to remove macrophages from the centers of *S. aureus* abscesses [139].

### Toxin-driven elimination of neutrophils

The mechanisms *S. aureus* uses to avoid killing by neutrophils that we have discussed so far are of what one could call “passive” in character. However, *S. aureus* also synthesizes a series of toxins that directly eliminate neutrophils and other leukocytes. These mainly consist of α-toxin (Hla) [140], the bicomponent leukocidins [141], and the PSMs [142].

Alpha-toxin is probably the most famous and also most important toxin of *S. aureus* in terms of contribution to pathogenesis (Figure 1). It has multiple functions; we have already discussed the role in epithelial breach in the previous section. Most notably, α-toxin is a major cytolysin for many cell types including leukocytes [140]. It is a 33 kDa protein in its mature form and oligomerizes into a heptameric structure that forms a stable membrane-spanning pore [143]. Pore formation is a receptor-dependent process that uses the ADAM10 protein as a receptor, explaining the differences in cytolytic capacity toward different cell types from different species that have been reported for α-toxin [32,140]. Alpha-toxin induces a series of inflammatory events in the target cell and induces the NLRP3 inflammasome, generally at lytic concentrations and likely forming part of the events leading to cell death by pyroptosis [140,144]; but some are also observed at sublytic concentrations [145]. The contribution of α-toxin to *S. aureus* infection has been demonstrated using isogenic mutants in many animal infection models, including pneumonia [146,147], skin infection [148], and sepsis [149], to name but a few.

The bicomponent leukocidins include Panton-Valentine leucocidin (PVL, encoded by the *lukS-PV* and *lukF-PV* genes), LukDE, LukAB (LukGH), and the HlgAB and HlgCB combinations of the HlgA, HlgB, and HlgC proteins (also called γ-toxin) [141]. All these toxins require assembly of a *lukF* and a *lukS* moiety. The S subunit recognizes a membrane protein receptor [a chemokine receptor for PVL and LukED [150,151] and an integrin for LukAB/GH [152]] and then recruits the F subunit, leading first to dimerization and ultimately, after considerable structural changes that facilitate membrane insertion, to the formation of an octameric pore [153]. All strains of *S. aureus* are capable of producing at least three (γ-toxin and LukAB/GH) leukocidins [141]. Highly virulent strains produce five, with PVL – which is only produced by an overall 2–3% of *S. aureus* isolates – having been in the center of attention due to its association with CA-MRSA isolates [154]. PVL has a considerable impact on CA-MRSA lung infection in a rabbit model [155], while its contribution to skin infection, the main manifestation of CA-MRSA disease, has remained controversial [148,156]. Notably, analysis of most leukocidins, with the noticeable exception of LukDE, is not possible in mouse models due to the species specificity of the leukocidin receptors [141], unless humanized mice are used [157]. The different receptors also underlie cell specificity, a likely reason for the fact that *S. aureus* produces several different leukocidins. All bicomponent leukocidins are quite specific for leukocytes, while γ-toxin also efficiently lyses erythrocytes by a receptor-mediated mechanism [158]. Of note, LukAB/GH is less related in sequence to the other leukocidins and appears to have specific functions. For example, it has been reported to facilitate lysis of targets cells after phagocytosis [159]. Finally, similar to α-toxin, leukocidins appear to have pro-inflammatory functions at sublytic concentrations, as shown particularly for PVL [160,161] .

PSMs are a family of amphipathic, α-helical peptides that include the early-described δ-toxin [142]. The α-type PSMs (~20–25 amino acids) have considerable cytolytic activity against neutrophils, which is most pronounced in the PSMα3 peptide [59]. The activity of PSMs is believed to be receptor-independent, leading to detergent-like membrane perturbation, and has been observed with many other cell types. At sublytic concentrations, PSMs have strong pro-inflammatory and chemotactic effects on neutrophils and keratinocytes [59,60,162]. PSMs have been reported to significantly contribute to blood [59] and lung infections [163], but there is epidemiological and experimental evidence indicating that their most pronounced relative contribution is to skin infections [59,164]. Of note, PSMs are – except possibly for LukAB/GH – the only leukocyte toxins that are believed to exert their main function after phagocytosis, and they have been shown to facilitate escape from the phagolysosome [165,166].

A more indirect way to eliminate neutrophils is to accelerate their natural self-destruction via apoptosis. Triggering of apoptotic events by *S. aureus* has been described for many toxins and cell types, such as by AdsA in macrophages [167], but specifically for neutrophils only in the case of PVL and the staphopain B protease [168,169].

## Tissue invasion

In systemic murine infection, *S. aureus* cells arrive soon (after 1–3 h) in the organs, where microscopically discernable lesions become visible after ~48 h [170,171]. Invasion by *S. aureus* of organs and tissues from the bloodstream not only requires immune evasion, as discussed above, but also adhesion and further structural processes. Many of these steps are facilitated by surface-anchored proteins that belong to the MSCRAMM (microbial surface components recognizing adhesive matrix molecules) family [172]. These proteins are secreted by the general secretion pathway and then tethered to peptidoglycan via sortase A-catalyzed reaction that links the threonine of a conserved N-terminal structure (LPXTG motif) to the amino group of the terminal glycine residue of the pentaglycine branch in peptidoglycan [173]. MSCRAMMs characteristically contain repeat sequences that allow spanning through the cell wall and exposed matrix protein-binding domains. Among the many MSCRAMMs with divergent functions, of which we discussed some involved in immune evasion already, the collagen-binding protein Cna and the fibronectin-binding proteins FnBPA and FnBPB have key functions in tissue adherence [174–176]. Providing evidence for the function of an MSCRAMM in its natural strain background is difficult due to functional redundancy. Such evidence has therefore in several cases been produced using knock-in (often by heterologous expression in *Lactococcus lactis*) rather than knock-out approaches [172].

## Maintenance of infection

### Abscess formation

Once an abscess is established, bacterial proliferation ensues in addition to the infiltration of a large number of leukocytes. Interestingly, in this situation, the main chemoattractants for leukocytes were shown to be lipopeptides, using a mutant in the *lgt* gene that codes for an essential lipoprotein synthesis enzyme. This mutant showed bacterial proliferation without typical abscess formation [177].

The altered scenario of high bacterial numbers surrounded by a high number of leukocytes requires considerable adaptation of bacterial physiology compared to the initiation of infection. First and foremost, mechanisms to evade killing by leukocytes, in addition to those discussed above, include the formation of an encapsulated abscess that inhibits additional infiltration of leukocytes [170]. One study used mutants in the cell wall-anchored proteins of strain Newman to monitor which factors promote which state of kidney abscess formation in a mouse systemic infection model. With the caveat that this strain does not produce or properly express some of the factors probably involved (such as Cna, FnBPA and FnBPB), it still gave key insight in abscess formation processes [171]. According to that study, iron (heme) acquisition facilitated by the IsdA and IsdB proteins is vital during the initial stage of abscess formation. The next stage of mature abscess formation is characterized by layers of necrotic and intact neutrophils surrounding the abscess center, which contains the proliferating *S. aureus* cells. In this stage, the coagulases Coa and vWbp produce fibrin clots to inhibit leukocyte infiltration. Another main contributor was protein A, although it is not known to which of protein A’s many functions the phenotype was due to [171]. Results from a more recent study indicate that the pro-inflammatory function of protein A is important for proper skin abscess formation and healing [178], which reflects, like many other studies, the importance of an adequate level of host response to *S. aureus* infection.

Furthermore, high bacterial density means that nutrients become scarce. *S. aureus* produces a series of cytolysins that can lyse cells and enzymes to digest the released nutrient macromolecules. In addition to the cytolytic properties of leukocidins toward leukocytes, we already mentioned the general cytolytic properties of PSMs, which may underlie the strong contribution those cytolysins have to the formation of subcutaneous, lung, and kidney abscesses [59,179,180]. Some *S. aureus* cytolysins synergize to achieve extremely strong hemolysis. This activity is known from the in-vitro effect known as CAMP reaction and occurs between β-toxin, a sphingomyelinase, and δ-toxin or other PSMs [181,182]. The main function of degradative exoenzymes, which include proteases, lipases, and nucleases, is assumed to be nutrient acquisition, but direct evidence is not available due to functional redundancy and the fact that many of these enzymes have additional functions, for example, in immune evasion.

### Biofilms

Another important way in which *S. aureus* maintains an infection is by the formation of biofilms [19]. These can form on abiotic material of indwelling medical devices, but also on tissue surfaces, such as on heart valves in the case of endocarditis. Biofilm formation develops in three main stages: adhesion, maturation/proliferation, and detachment. Adhesion in vivo occurs to human matrix proteins via cell-wall anchored and other surface proteins, many of which belong to the MSCRAMM family [172]. In the second stage, a biofilm matrix is produced that connects cells and which in *S. aureus* consists of the PIA/PNAG exopolysaccharide [83], extracellular DNA [183], teichoic acids [184] and – often amyloid-forming – proteins such as SasG [185]. Biofilms have a distinct three-dimensional structure with channels that are formed by the surfactant activity of PSMs and degradative exoenzymes such as proteases [186–188]. Detachment of cells clusters from the biofilm occurs by extensive activity of these biofilm-structuring factors.

The main role of biofilm formation during infection is to protect the bacteria from phagocyte attacks [189]. Some have argued that biofilm formation and abscesses have similar characteristics. However, there are certainly key differences. For example, biofilms are not usually surrounded by large layers of neutrophils as abscesses are, which is likely due to the fact that in biofilms, *S. aureus* lives in a comparatively less aggressive state [190] and does not produce or shed through the biofilm matrix a large amount of chemoattractant molecules. In addition, *S. aureus* biofilms have been shown to skew the host immune response toward an anti-inflammatory state [189,191].

## Internalization, persistence, and distribution of infection

A further way to hide from attacks of the immune system that *S. aureus* employs is to shelter within host cells. This has been shown for phagocytes such as neutrophils and monocytes [192–194] as well as a series of non-phagocytic cells, including epithelial and endothelial cells, keratinocytes, and osteoblasts [195]. *S. aureus* that persists in neutrophils is infectious and this mechanism is believed to contribute to the spread of *S. aureus* during an infection [193]. Most likely, its main role is in persistence during passage through the bloodstream. Invasion of non-phagocytic cells contributes to chronicity of infection [195] and is mediated in part by FnBPs [196], but also other factors such as Eap [197]. FnBPs bind fibronectin on the cell surface via a tandem-β-zipper mechanism [198]. After internalization by phagocytosis or via FnBPs, *S. aureus* escapes from the phagosome, involving the abovementioned activity of PSMs [199]. The events that lead to intracellular persistence versus target cell lysis are incompletely understood. Formation of so-called small-colony variants (SCVs), which are cells with reduced metabolism that do not express cytolysins, are likely involved [200]. It is interesting that SCVs express high amounts of FnBPs, facilitating invasion of neighboring cells upon cell lysis [201].

While persistence in phagocytes may have a role in spreading through the bloodstream, escape from an established infection site, such as an abscess or a biofilm, requires specific further mechanisms. Factors that rupture an abscess and lead to local dissemination include staphylokinase, an enzyme that forms a complex with plasmin, catalyzing further plasminogen activation, which ultimately leads to increased proteolysis and fibrinolysis [202]. Of note, the activity of staphylokinase is specific for human plasminogen. Systemic spread from an established in-vivo biofilm infection is mediated by PSMs, which facilitate biofilm detachment [186]. It may hypothetically also occur via biofilm-degrading enzymes [188]; yet this has not been established in vivo.

While initiation and maintenance of infection during the first days is largely due to efficient evasion of innate immune defenses, the interaction of *S. aureus* with the acquired immune defense becomes defining once this arm of the immune system is activated, after about one to two weeks post-infection. We already mentioned the crucial importance of protein A in that aspect and the many ways by which *S. aureus* interferes with opsonization. Another way by which *S. aureus* interferes with the adaptive immune system is by subverting T-cell responses, particularly of IL-17-producing T-cells, which many lines of evidence suggest have a key role in host defense against *S. aureus* [203,204]. *S. aureus* may induce a status of adaptive tolerance in T-cells (in-vivo energy), for example, via enterotoxin B [205], and superantigens, in general, may reduce protective T-cell responses [206]. Furthermore, the inhibition of T-cell responses that is observed during murine *S. aureus* infection [207] occurs via myeloid-derived suppressor cells (MDSCs), and to a minor extent regulatory T-cells (Tregs) [208], but it is not known how *S. aureus* induces this mechanism.

## Genetics of virulence

Virulence factors of *S. aureus* are often encoded on the pathogen’s accessory genome that differs from the core genome, which predominantly encodes “housekeeping” functions. The accessory genome contains mobile genetic elements (MGEs) like plasmids, transposons, insertion sequences, prophages, and pathogenicity islands, which in addition to virulence factors also contain antibiotic resistance determinants [209,210]. The large family of staphylococcal pathogenicity islands (SaPIs), while not containing transfer machinery like plasmids or phages, rely on helper phages for transduction [211]. The accessory genome also contains genomic islands (νSAα, νSAβ, νSAγ), which encode a series of virulence factors and appear to have originated from MGEs, but lost the ability to be transferred other than by non-MGE-specific modes of transfer. They are thus quite stable and so widespread that their contents can be considered characteristic for the entire species, although specific subtypes are associated with different lineages [212]. This contrasts the isolate-specific MGEs, which are often linked to specific diseases (“toxinoses”) due to the encoding of the respective responsible toxins, such as TSST-1 or the food poisoning enterotoxins [210,211].

Plasmids and transposons typically contain antibiotic resistance genes, while phage-related and pathogenicity islands contain most *S. aureus* toxins and other virulence determinants [210]. Important *S. aureus* toxins encoded on prophages include PVL, the immune evasion proteins CHIPS and SCIN, the exfoliative toxins A and B, as well as staphylokinase and a series of enterotoxins. Of note, the gene encoding β-toxin (β-hemolysin), *hlb*, which has been associated with virulence functions [213], is rendered nonfunctional in many *S. aureus* strains by insertion of the phage that carries CHIPS, SCIN, and staphylokinase [214], a process generally called “negative conversion.” There is evidence that *hlb* may be “repaired” by phage excision and is important for infectious colonization [215]. SaPIs are mostly known for enterotoxins and TSST. Toxins that are encoded on genomic islands and usually only vary in expression between different isolates include α-toxin, PSM peptides, SSLs, the lipoprotein-like toxins (LPLs), the leukocidin LukDE, and some enterotoxins [210,216,217]. Interestingly, the genomic island νSAβ also contains what appears to be an intact biosynthesis cluster for the production of a lantibiotic, but its expression and potential role in bacterial interference has never been directly shown [218]. Furthermore, it needs to be stressed that while many MSCRAMMs have key roles in virulence, they are usually not encoded on the accessory genome, probably as they have general functions in the commensal lifestyle of *S. aureus*.

## Regulation of virulence

**Figure 4.**
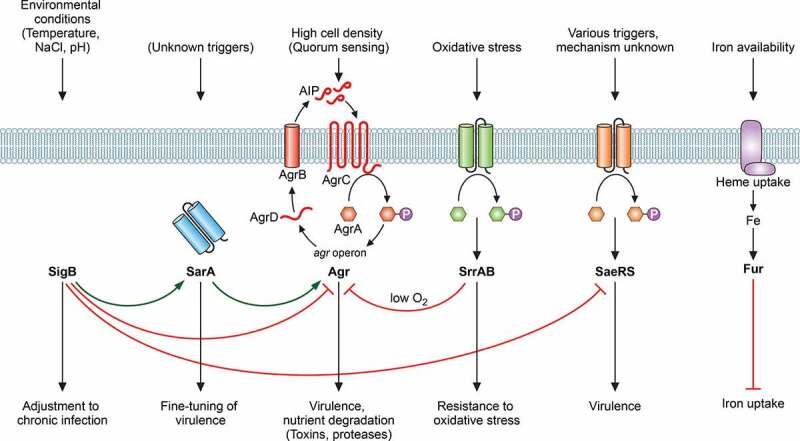
Regulation of virulence in *S. aureus.*

The expression of *S. aureus* virulence determinants is subject to a wide variety of regulatory influences (Figure 4). These include regulation by locus-specific regulatory factors, such as the *icaR* gene adjacent to the *ica* operon [219], which is itself subject to many regulatory impacts [220], or the PSM-sensing PmtR protein controlling the PSM exporter Pmt operon [58,221], as well as global regulators that regulate a series of virulence genes and which are often driven by specific environmental conditions. Here, we will focus on some selected, key global regulators.

Virulence regulation in *S. aureus* is highly complex and mediated by a large amount of regulatory systems. Only the most important are depicted here. Many virulence factors are impacted by the alternative sigma factor SigB, the Sar family of DNA-binding proteins (of which SarA is shown), the Agr quorum-sensing system, and further regulators such as SaeRS. Except for Agr, the exact triggering mechanisms of these systems remain largely elusive. Oxygen is sensed by SrrAB, which has an important role in ROS resistance, and Fur is a DNA-binding repressor that regulates iron uptake, but also virulence genes.

The most extensively studied staphylococcal virulence regulator is Agr (accessory gene regulator), which is a quorum-sensing system that up-regulates many toxins and virulence determinants when cell density reaches a certain threshold [222,223]. This regulation is believed to link the expression of virulence determinants to the state of infection when they are needed for nutrient acquisition and immune evasion, while delaying their expression during early infection to prevent the triggering of immune responses. Furthermore, within a phagosome, Agr-controlled virulence factors are expressed presumably because the confined environment activates the quorum-sensing system by a mechanism called “diffusion sensing” [224]. Historically, Agr has been described to generally down-regulate adhesion factors, whose main function was supposed to be during early infection, but this notion has mostly been reversed due to the recognition of many additional functions of MSCRAMMs and the finding that Agr does not regulate most MSCRAMMs in such fashion in clinical strains [225]. Most stringently controlled by Agr are secreted proteases and the PSMs. PSMs are directly controlled by the response regulator of the Agr two-component system, AgrA, while other Agr targets are regulated in an indirect fashion via the regulatory RNAIII, which forms part of Agr and represents its main intracellular effector molecule [226,227]. RNAIII-dependent gene regulation occurs in most cases via inhibition of a DNA-binding repressor protein called Rot [228]. The extracellular quorum-sensing signal of the Agr system is a post-translationally modified short thiolactone-containing autoinducing peptide (AIP) [229]. *S. aureus* has at least four Agr subgroups that differ in the sequence of the AIP, its modifying enzyme AgrB and the membrane histidine kinase AgrC, to which it binds and which upon activation triggers phosphorylation of AgrA [230]. Most AIPs of non-self, including those from other staphylococcal species, are inhibitory, which may play a role in interbacterial interaction in vivo [231,232]. As expected from the regulation of many toxins and other virulence factors, *agr* mutants show significant defects in many animal infection models, such as in infective endocarditis [233], skin and soft tissue infections [225,234], pneumonia [235,236], septic arthritis [237], osteomyelitis [238], and atopic dermatitis [239].

Mutants in *agr* arise frequently in a process known as “quorum cheating,” which describes the situation that specific members of a population mutate *agr* to save energy and benefit from the maintained Agr function of other cells in the population [240–242]. Populations only or predominantly consisting of Agr-dysfunctional cells have benefits in biofilm-associated infections due to the enlarged biofilms that *agr* mutants form and the concomitant increased resistance toward neutrophil attacks [242]. This is believed to explain the increased frequency of *agr* mutants isolated from chronic infection and bacteremia [243], which commonly originate from biofilm-associated infection of indwelling medical devices. Of special note, spontaneous mutation in *agr* occurs frequently in the laboratory, probably due to the absence of selective pressure for virulence factor expression, which – together with a lack of thorough genetic analysis – can lead to erroneous attribution of regulatory functions to other proteins and systems [244–246].

Further important global regulators for which the inducing environmental cues are known include Fur, which responds to low iron availability [247], and SrrAB, which is an oxygen-responsive regulator [248]. Fur not only regulates iron uptake, but also a series of virulence factors such as toxins and immune evasion proteins [249–251]. The role of Fur is believed to consist in coordinating the pathogen’s attack on the host, with iron restriction signaling entry into the body and consequent need for those factors. Accordingly, *fur* mutants show significant reduction in virulence in animal infection models [250,251]. SrrAB is an oxygen-sensitive two-component system that relies on redox-sensitive cysteines [252] to promote resistance to oxidative stress [253]. Under anaerobic conditions, it down-regulates *agr* while up-regulating *ica* expression, with the consequence of increased resistance to neutrophil attacks [254].

The Sar family comprises several short proteins (~120 amino acids) with helix-turn-helix DNA-binding sequences [255]. They are all homologous to the SarA prototype and believed to form one or two-domain dimeric winged helix structures [256]. Sar family proteins have multiple virulence factor targets and interact with a multitude of other regulatory systems [255]. Often the impact of one Sar homolog on a given virulence factor gene can be opposite to that of another. SarA is mostly known for its strong impact on protease expression, which is achieved in part but not entirely through regulation of Agr [257]. Although it is believed that the reason for the existence of multiple Sar homologs is to fine-tune virulence factor expression according to different environmental conditions, similar to many other regulators with pronounced and divergent impact on the expression of *S. aureus* virulence determinants, such as SaeRS [258] and ArlRS [259], the molecular or environmental triggers of many Sar family regulators are not known [255]. A noticeable exception is MgrA, which has multifold effects on virulence [260–263], contains redox-sensitive cysteines and thus reacts to oxygen and reactive oxygen species [264]. MgrA is similar to SarZ, which also works as an oxidation sensor using thiol-based oxidation sensing [265].

Similar to many other bacteria, *S. aureus* has an alternative sigma factor called SigB, whose gene is embedded in a locus also harboring a series of anti-sigma factor genes, which together are believed to react to a multitude of environmental conditions such as growth phase and heat shock [266]. SigB interacts with many other regulators, such as Agr and SarA, and has a profound impact on virulence gene expression [267,268]. It is believed to adapt *S. aureus* physiology to chronic infection [269].

## Anti-virulence therapeutic strategies

Driven by the concerning global spread of antibiotic resistance, and in the case of *S. aureus* the additional, ongoing difficulties of finding a working vaccine, there is increased recent interest in the development of alternative treatment strategies for bacterial infections. Anti-virulence strategies, which represent the translational arm of bacterial pathogenesis research, are often claimed to have a smaller risk for the development of resistance, but in *S. aureus* face the considerable problem of multiple and often functionally redundant virulence factors [270]. Therefore, anti-virulence approaches in *S. aureus* either target a virulence determinant with established widespread and extraordinarily important impact on pathogenicity, or aim to eliminate several virulence factors at a time. Three main *S. aureus* anti-virulence approaches that are currently being investigated follow one or both of these strategies. There is first the development of monoclonal antibodies against α-toxin, which in many isolates is a predominant virulence determinant, such as MEDI4893 (suvratoxumab) developed by MedImmune [271,272]. Second, several approaches aim to target all *S. aureus* leukocidins, often in addition to the somewhat similar α-toxin. This approach has been taken for example by the Austrian company Arsanis, who developed a mAb with cross-reactivity against all leukocidins and α-toxin [273,274]; however, clinical trials have failed. Third, quorum-sensing blockers targeting Agr have been promoted by many researchers [275]. They mainly comprise two classes: those that interfere with AIP binding to AgrC from the extracellular space, such as AIP analogs [276] and several natural compounds including fengycins [277], and those that need to penetrate into the cytoplasm to inhibit the response regulator AgrA, and are commonly more hydrophobic, such as savirin or apicidin [278,279]. Notably, Agr inhibitors have not yet been analyzed using rigorous testing with systemic application in models of the types of systemic disease for which anti-virulence drugs would be most desirable. Furthermore, they would likely have to be limited to acute types of infection, while for chronic and especially biofilm infection they may be counterproductive [275]. Moreover, as for all anti-virulence compounds, it needs to be established that their in-vivo efficacy is in fact due to their anti-virulence rather than bactericidal effects, which many of especially the more hydrophobic compounds show at only slightly higher concentrations.

## Outlook

There has been a considerable recent increase in our knowledge about *S. aureus* pathogenesis and *S. aureus* virulence determinants. In addition to many open mechanistic questions, main current challenges are (i) how to use the gained understanding for the development of anti-virulence drugs, and (ii) how to integrate those findings into those from the newly developing field of bacterial interactions in the human microbiome. A particular technical problem arises from the host specificity of several *S. aureus* virulence factors, such as the bicomponent leukocidins, calling for the increased use of humanized mice and/or inclusion of other species in *S. aureus* virulence research.

As for the translational use of virulence findings, it will be crucial to rank the importance of virulence factors depending on strain and disease type in order to select those against which a multi-pronged therapeutic should be developed. Most previous virulence studies focus on one factor, for which a significant role in a given animal infection model is presented; but only very few studies have tried to directly compare different factors using experimental approaches [148,280], especially for the purpose of drug development [281]. Such endeavors have already been taken comparing the impact of different cytolysins on the virulence of important CA-MRSA lineages [179,280], but they need to be considerably expanded in order to base drug development strategies, such as for the development of cross-reactive mAbs or mAb cocktails, on research findings rather than educated guesses. Genetic tools to produce deletion mutants in the virulence genes of interest in clinical strains have been developed [282,283] and this should therefore not represent a bottleneck for such research anymore. Except for mAbs against leukocidins and α-toxin, all anti-virulence approaches are still at the investigational state. Quorum-sensing blockers and other investigational anti-virulence compounds need to undergo more rigorous pre-clinical testing using the established standards for drug development.

*S. aureus* pathogenesis research is also expected to receive substantial stimulation in the years to come from the field of microbial interactions, which has experienced a recent revival due to the ability of in-depth microbiome analyses. Already, interactions have been identified that alter *S. aureus* virulence factor expression, particularly via inhibition of the Agr system, by co-colonizers [232,277]. These findings may lead to the discovery of new drugs or even probiotic approaches for anti-virulence strategies against *S. aureus*.
